# Giant scrotal lymphedema of unclear etiology: a case report

**DOI:** 10.1186/1752-1947-3-7295

**Published:** 2009-05-28

**Authors:** Ganiyu A Rahman, Ismaila A Adigun, Ibrahim F Yusuf, Adebiyi B Aderibigbe, Amarachukwu C Etonyeaku

**Affiliations:** 1 Division of General Surgery, Department of Surgery, University of Ilorin Teaching Hospital, Ilorin, Nigeria; 2 Division of Plastic and Reconstructive Surgery, Department of Surgery, University of Ilorin Teaching Hospital, Ilorin, Nigeria

## Abstract

**Introduction:**

Scrotal lymphedema is common in the tropics and subtropics. The giant variants can cause a lot of physical disability and psychological disturbances.

**Case presentation:**

We present a 25-year-old Nigerian male with giant scrotal lymphedema with severe debilitating symptoms, immobility and emotional disturbance. He benefited from a modified Charles' procedure and reconstruction of the penile shaft using a split-thickness skin graft.

**Conclusion:**

Giant scrotal lymphedema related to poverty, ignorance and neglect, is amenable to surgery. Surgery provides a cosmetically acceptable and functionally satisfying outcome.

## Introduction

Giant scrotal lymphedema, also known as elephantiasis, can be caused by obstruction, aplasia or hypoplasia of lymphatic vessels [1]. Though it can be caused by a neoplasm, radiotherapy or lymphadenectomy, most cases are usually caused by infection as a result of lymphogranuloma venereum or filarial infestation with *Wuchereria bancrofti*[1-3]. Giant scrotal lymphedema is common in the tropics and sub-tropics [4,5].

We present our experience of treating an unusually large penoscrotal lymphedema and the result of a modified Charles' procedure and cutaneous reconstruction of the penile shaft using a split-thickness skin graft.

## Case presentation

A 25-year-old single Nigerian man was admitted via the surgical outpatient clinic with a two-year history of scrotal swelling which was initially small in size, non-painful and not associated with fever. The swelling gradually increased in size to the extent of impairing free movement of the patient both because of its weight and size in between the lower limbs. The swelling made sexual intercourse and voiding in the standing position impossible. The skin was intact without associated ulcerations, and there was no difficulty in voiding though the penile shaft was buried in the swelling. There was no history of chronic cough suggestive of pulmonary or disseminated tuberculosis. He had no problems with his eyesight and no other swellings on his body were known.

He had a history of surgery to the two inguinal areas about 10 years before presentation; surgical details were not available nor was any histology available. He was from a polygamous home and took neither alcohol nor any other substance of abuse. There was no history of a similar lesion in his relatives or his acquaintances at his place of abode.

**Figure 1:**
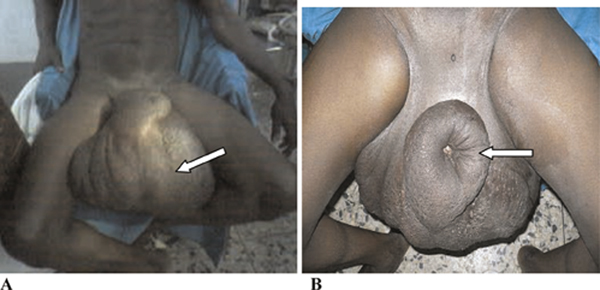
**Photograph of a 25-year-old man showing giant scrotal lymphedema; (A) scrotal lymphedema (arrowed) and (B) the buried penis with external meatus as a dimple (arrowed)**.

Physical examination showed a healthy looking young man with a giant scrotal swelling of a size greater than that of his head. There were hyperpigmented giant ruggae in otherwise intact skin with the penile shaft buried in the scrotal wall skin (Figure 1a and 1b). The swelling was non-tender, non-pitting and non-reducible with the cord barely discernible at the neck. Bilateral inguinal scars were seen with significant lymphadenopathy.

Hemogram, serum electrolyte, urea and creatinine, urinalysis, urine microscopy culture and sensitivity were all within normal limits. Scrotal ultrasound showed a thickened scrotal wall but the scrotal content was normal.

**Figure 2:**
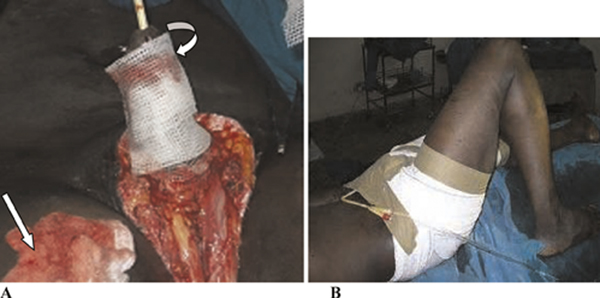
**Intra-operative and immediate postoperative photographs of the patient; (A) the donor site (straight arrow) and the penile shaft sofratulle dressing (curved arrow); (B) postoperative dressing with the urethral catheter anchored to the anterior abdominal wall**.

He subsequently underwent a modified Charles' procedure with a primary penile shaft split-thickness skin graft (Figure 2A) performed by a team of general surgeons and plastic surgeons. His immediate postoperative condition was satisfactory (Figure 2B). On the postoperative day seven, the penile skin graft showed good skin take and some good granulation tissue over the exposed testicles.

**Figure 3:**
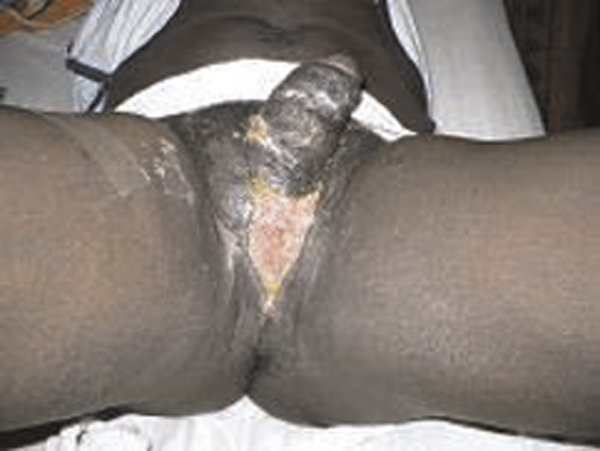
**Photograph showing the appearance of the external genitalia and healed donor site six weeks after the operation**.

The patient continued to have wound dressing until the perineal wound had healed and contracted appreciably leaving about a 3 cm raw area of the wound at the six postoperative week (Figure 3). He had an uneventful postoperative course with the scrotal wound healing completely by the eighth postoperative week (Figure 3). He is now about one year post-surgery and doing well. The patient is physically and socially satisfied with his improved quality of life.

## Discussion

Lymphedema of the penis and scrotum is due to abnormal accumulation of lymphatic fluid in subcutaneous tissue of the penis and scrotum. Lymphedema has two types: primary and secondary. Primary lymphedema can be congenital-inherited (Milroy's), praecox or tarda. Secondary lymphedema has three origins: obstructive (secondary to neoplasm, radiotherapy, surgical intervention, mechanical trauma, bacterial and fungal infections), phlebitial or angioneurotic [6]. Penoscrotal lymphedema mostly occurs following an infection or as a reaction to trauma. Scrotal lymphedema can be emotionally distressing and physically disabling. It is a condition leading to progressive enlargement of the scrotum and penis. In addition to the grotesque aspect, the progression of the condition impairs ambulation, makes sexual intercourse impossible, and impairs both voiding in the standing position and proper hygiene of the perineal region, with subsequent malodor and recurrent episodes of skin infection, all causing severe damage to the patient's quality of life and self-esteem [7-9].

Our 25-year-old patient had scrotal swelling for about two years. At the time of presentation, he had a giant scrotal swelling with buried penis. This was probably a result of lymphatic obstruction of the superficial chain of the inguinoscrotal region, responsible for the lymphatic drainage of the penile and scrotal skin. Though this patient has lived in an endemic region, there was no evidence of filariasis. This is difficult to explain. The etiological factor in this patient could not be clearly identified. A possibility is the previous inguinal operation, the details of which were not known. It could have been due to hernial surgery or lymphadenectomy.

The use of non-surgical treatment with different approaches has proven inefficient and is not longer used in large edemas [8,10]. Surgical intervention was therefore inevitable in this patient. Our patient had complete excision of all elephantoid tissue of the scrotum and penile skin with saving of the penis, spermatic cord and testes which is the recommended treatment for such a massive scrotal elephantiasis [5].

There are two main methods of surgical treatment of chronic genital lymphedema: lymphangioplasty and lymphangiectomy with reconstructive surgery [10]. Where chronic fibrosis has led to lack of appropriate lymphatic channels, lymphangioplasty is not feasible or successful. Lymphangiectomy includes the removal of the superficial lymphatic network derived from the median raphe and prepuce lymphatics which is located above Buck's fascia. This lymphatic network drains into the superficial posterior lymphatic channel. A deeper system is located beneath Buck's fascia and drains into the deep inguinal lymph nodes [6]. It is important to remove involved skin and subcutaneous tissue completely to prevent recurrence.

The major challenge in this procedure is the reconstruction of the penile skin. There are various procedures in the surgical literature to achieve this [7,8]. We opted for the use of a split-thickness skin graft. This seems to produce better results than the use of flaps, even local flaps which notably affect tactile sensitivity and erection [9]. The outcome of this procedure has been very satisfactory. There is a good cosmetic result for the external genitalia with easier ambulation, effective hygiene, voiding and subjectively satisfactory sexual intercourse. The histopathological section showed skin tissue with papillomatosis. The dermis showed dense lymphoplasmocytic infiltration and dense fibrocollagenous tissue with dilated lymphatic channels. These features were consistent with elephantiasis. One year after the procedure, the patient has done very well with improvement to his quality of life.

## Conclusion

Massive penoscrotal lymphedema can cause physical disability, social embarrassment and can be psychologically disturbing. The uniqueness of this case is the unclear etiology and achieving complete surgical cure without a rotational flap and vacuum assisted devices recommended in such a large scrotal lymphedema. The patient benefited from a modified Charles' procedure with a primary penile shaft split-thickness skin graft and achieved a satisfactory outcome.

## Consent

Written informed consent was obtained from the patient for publication of this case report and any accompanying images. A copy of the written consent is available for review by the Editor-in-Chief of this Journal.

## Competing interest

The authors declare that they have no competing interests.

## Authors' contribution

All authors were actively involved in the management of this patient. They also participated in the design and writing of the manuscript and all have read and approved the final version.
